# Longitudinal Influences of Neighbourhood Built and Social Environment on Children’s Weight Status

**DOI:** 10.3390/ijerph10105083

**Published:** 2013-10-15

**Authors:** Maria Gose, Sandra Plachta-Danielzik, Bianca Willié, Maike Johannsen, Beate Landsberg, Manfred J. Müller

**Affiliations:** 1 Institute of Human Nutrition and Food Science, Christian-Albrechts University Kiel, Düsternbrooker Weg 17, Kiel 24105, Germany; E-Mails: gose.maria@gmx.de (M.G.); sdanielzik@nutrfoodsc.uni-kiel.de (S.P.-D.); mjohannsen@nutrfoodsc.uni-kiel.de (M.J.); blandsberg@nutrfoodsc.uni-kiel.de (B.L.); 2 Centre for Geoinformation, Christian-Albrechts University Kiel, Neufeldtstraße 10, Kiel 24118, Germany; E-Mail: bwillie@torresin-und-partner.de

**Keywords:** overweight, children, longitudinal study, neighbourhood environment

## Abstract

The objective was to examine longitudinal 4-year-relationships between neighbourhood social environment and children’s body mass index-standard deviation score (BMI-SDS) taking into account the built environment. Furthermore, we have analysed the influence of potential interactions between the social environment and family/social data on children’s BMI-SDS. Between 2006–2008 and 2010–2012, anthropometric measurements were conducted among 485 children (age at baseline: 6.1 (5.8–6.4)). Socio-demographic characteristics and perception of residential environment were reported by parents. Geographic Information Systems were used to examine street length, number of food outlets and distance to the nearest playground and park/green space within an 800 m Euclidian buffer of each participant address point. Additional data on neighbourhood characteristics (e.g., traffic density, walkability, crime rates) were obtained from the State Capital of Kiel, Germany. In a multivariate model, walkability, street type, socioeconomic status of the district and perceived frequency of passing trucks/busses were associated with BMI-SDS over 4 years, but only neighbourhood SES had an effect on change in BMI-SDS. However, familial/social factors rather than neighbourhood environment (especially social environment) had an impact on children’s BMI-SDS over 4 years. Thus, social inequalities in childhood overweight are only partially explained by social neighbourhood environment.

## 1. Introduction

Besides biological factors, individual behavior as well as familial and social factors add to explain childhood obesity [1]. However, human ecological approaches assume that human behaviour is not only influenced by individual and interpersonal factors but also by interactions with the environment [2]. Related to obesity, the environment can be classified into macroenvironmental sectors (e.g., food industry, media and health system) and microenvironmental settings (e.g., homes, schools and neighbourhoods) [3]. In recent years, the number of studies exploring the association between obesity and the microenvironmental setting “neighbourhood” (geographic area where residents share proximity and circumstances [4,5]) has grown rapidly. These studies suggested that the neighbourhood environment affects energy balance by facilitating and hindering physical activity and healthy eating. For example, Davison and Lawson observed a higher physical activity among children living near parks and playgrounds [6]. In addition, Timperio *et al*. and Oreskovic *et al*. found that a high density of fast food outlets was associated with a low fruit intake and a high body mass index (BMI) [7,8]. On the other hand, other authors could not find any statistical relationships between the neighbourhood environment and children’s overweight, diet and physical activity, respectively [9,10,11,12]. 

Nevertheless, the existing research has tended to focus on the influence of the built environment on obesity, nutrition and physical activity behaviour. The term “built environment” refers to human-made or modified surroundings, like buildings, land use or green spaces [3,13]. However, the built environment is only one environmental dimension. In addition to the political and economic environment, the social environment represents another possible dimension which includes factors such as crime rates, neighbourhood socioeconomic status, social cohesion and network [3,14]. Unfortunately, little attention has been paid to the social environment in recent literature. In Germany, Lange *et al*. indicated that adolescents living in neighbourhoods with high unemployment rates spent more time in front of a computer or television and more often showed an unhealthy eating pattern than adolescents living in neighbourhoods with low unemployment rates, whereas a high number of parks and sport fields were inversely related to high media time and unhealthy eating pattern [15]. Furthermore, Igel *et al*. demonstrated that a high deprivation rate of the district was independently associated with overweight among pre-school children [16]. These studies gave evidence that inequalities in neighbourhood social environments may add to overweight among children.

However, most studies mentioned above were based on cross-sectional data and most of the longitudinal studies had only concentrated on social characteristics of the neighbourhood environment excluding parameters of the built environment [17,18]. Therefore, the purpose of this study was: (i) to examine the longitudinal relationships between social neighbourhood environments and children’s BMI standard deviation score (BMI-SDS) over 4 years taking into account the built neighbourhood environment and (ii) to analyse the longitudinal influence of potential interactions between the social neighbourhood environment and family/social data on children’s BMI-SDS.

## 2. Methods

### 2.1. Participants

Data were collected as part of the Kiel Obesity Prevention Study (KOPS), which is an ongoing study started in 1996 in Kiel (Northern Germany). The aims and details of the study design have been described elsewhere [19]. The study population of this paper is based on 2,767 children (age: 5–7 years) who were recruited during school entry examinations between 2006 and 2008. A total of 758 of our initial population (27.4%) could be re-investigated at the age of 9–11 years (2010–2012). There were no significant differences in baseline BMI-SDS and socio-demographic parameters between children who participated in follow-up and children who did not. Further, only data of children who: (i) had a valid residential address in Kiel, (ii) did not change residential address during this time period and (iii) whose parents completed a questionnaire on social data, household characteristics and neighbourhood perceptions at follow-up (2010–2012) were included in the analysis (*n* = 485, 17.5%; excluded: (i) *n* = 18, (ii) *n* = 83, (iii) *n* = 172). Children who were excluded from analysis had a higher BMI-SDS at baseline and had more often a “non-German” nationality and low educated parents compared with children who were included.

This study was conducted according to the guidelines laid down in the Declaration of Helsinki and all procedures involving human subjects were approved by the local ethics committee of the Medical Faculty of the Christian-Albrechts University, Kiel. Written informed consent was obtained from all families before participation. 

### 2.2. Weight Status

Anthropometric measurements were conducted at baseline and follow-up. Children’s height and weight were measured in underwear or light clothing without shoes using a portable stadiometer (Model 214; Seca Vogel and Halke, Hamburg, Germany) and a calibrated digital scale (Model 861; Seca Vogel and Halke). Body Mass Index (BMI) was calculated (kg/m^2^) and the 90th age and sex-specific German BMI percentile was used to define overweight including obesity [20]. Individual BMI data were converted to BMI-SDS. The calculation of BMI-SDS (=outcome) was based on national reference data for German children [20]. Maternal weight and height were self-reported. WHO cut-offs were applied to categorise parental BMI (overweight inclusive obesity ≥ 25 kg/m²) [21].

### 2.3. Social Factors

The educational level of the family was based on parents’ years of schooling and was classified into three categories: “low” ≤ 9 school years, “middle” = 10–11 school years and “high” ≥ 12 school years (highest level attained by either parents). Nationality was determined on the basis of parents’ country of birth (“German” and “non-German”). Families with one “German” and one “Non-German” parent were excluded.

### 2.4. Built Environment

Data of the built environment were only assessed at follow-up. A Geographic Information System (Arc GIS^®^ Arc MapTM 10.1; Esri Inc., Redlands, CA, USA) was used to generate objective measures of street length, number of food outlets and distance to the nearest playground and park/green space within an 800 m Euclidian buffer of each participant address point (equating to a 10 min walk). In 2012, all food outlets of Kiel were visited on foot and geocoded using GPS. In addition, type of the food outlet was assessed (supermarket or energy-dense food supply like fast food outlet, takeaways, petrol station, *etc*.). Data of street length, playgrounds and parks/green space were supplied by the State Capital of Kiel for the year 2012.

Data of traffic density of each district of Kiel was provided by the Kiel Civil Engineering Office. Number of cars per day registered in several street stations was referred to the area (0.01 km^2^) of each district. 

Based upon the procedure described by Frank *et al.* [22], walkability of each district was calculated using an index which was the sum of weighted z-scores of population density (number of households/district), road connectivity (intersection density) and land-use mix.

In addition to objectively measured parameters, we simultaneously assessed parents’ perception of their neighbourhood built environment by means of the follow-up questionnaire. Parents were asked to indicate the existence of a garden, the type of house and street in which they live, the frequency of traffic jam and passing trucks or buses and if they feel restricted, for example, by the lack of sidewalks, cycling path or playgrounds/parks.

### 2.5. Social Environment

Neighbourhood socioeconomic status (SES) was derived from 2007–2011 district data (Kiel Statistics Department). To characterise neighbourhood SES, three variables were selected: unemployment rate, proportion of immigrants and percentage of welfare recipients within the specific neighbourhoods. Average crime rates (2007–2011) were calculated for each district and related to average number of inhabitants. Furthermore, parents were asked to report if they feel safe in their neighbourhood and if they are satisfied with their neighbourhood. 

### 2.6. Statistical Analysis

Statistical analysis was performed with IBM^®^ SPSS^®^ Statistics 20 (Armonk, NY, USA). Results are presented as median and interquartile range (IQR). Non-parametric Mann-Whitney U test was used to detect differences in continuous variables between baseline and follow-up. Chi-squared test compared the prevalences of categorical variables. Ordinal regression was used to identify associations between ordinal scaled variables of the neighbourhood social environment and socio-demographic factors.

The longitudinal relationship between neighbourhood built and social environment and BMI-SDS was analysed using generalized estimating equations (GEE). GEE represents an extension of the generalized linear model by providing support for correlated data, such as repeated measures. In GEE, between-subject and within-subject correlations are taken into account resulting in a single regression coefficient. The regression coefficients have a “population-averaged” interpretation. First, the bivariable relationship between each predictor and BMI-SDS was established (Model 1). Model 2 (multivariate analysis) included all predictor variables which were related to BMI-SDS at *p* < 0.05 in Model 1 and their time interaction term. The corrected quasi-likelihood under the Independence Model Criterion (QICC) statistic was used to assess model fit. If inclusion of an interaction term resulted in a smaller QICC value, the interaction term was retained. In the final model (Model 3), results of Model 2 were adjusted for children’s sex, maternal weight status and educational level and nationality of the family. In addition, possible interactions between socio-demographic factors and environmental predictors were taken into account. Level of significance was set at *p* < 0.05 (two-sided).

## 3. Results

Descriptive statistics of the study populations and the neighbourhood built and social environment are presented in Table 1 and Table 2. There were no sex differences in the anthropometric and social data. 

**Table 1:** Characteristics of the study population.

				Longitudinal study (*n* = 485)	
				Baseline	Follow-up
**Age (y)**				**6.1** (5.8–6.4)	**10.0** (9.7–10.3)
**Boys/girls (% (95% CI))**				50.5 (46.1–54.9)/49.5 (45.1–53.9)	
**BMI (kg/m^2^)**				**15.5** (14.6–16.5)	**17.1** (15.7–19.0)
**BMI-SDS**				**0.048** (−0.540–0.567)	**0.111** (−0.606–0.786)
**∆ BMI-SDS**				−0.006 (−0.375–0.460)	
**Prevalence of overweight (% (95% CI)) ^1^**				8.2 (5.8–10.6)	12.3 (9.4–15.2)
**Prevalence of maternal overweight (% (95% CI)) ^2^**				*n* = 467	
				35.1 (30.8–39.4)	
**Educational level of parents (% (95% CI))**				*n* = 478	
			High	59.8 (55.4–64.2)	
		Middle		29.3 (25.2–33.4)	
		Low		10.9 (8.1–13.7)	
**Nationality (% (95% CI))**				*n* = 478	
	“German”			86.0 (82.9–89.1)	
	“Non-German”			14.0 (10.9–17.1)	

BMI: body mass index; SDS: standard deviation score; ^1^ Kromeyer-Hauschild *et al*. (2001); ^2^ WHO (2000); Results are presented as median and interquartile range (IQR) or prevalence and 95% confidence interval (CI). Marked bold: significant differences between baseline and follow-up (Mann-Whitney U test; *p* < 0.001).

### 3.1. Characteristics of Socially Disadvantaged Neighbourhoods

Districts with a low SES had more often a high crime rate than their counterparts with a high SES (14.3% *vs*. 0%, *p* = 0.004). In addition, number of supermarkets (ß = 1.8; *p* = 0.000) within a 800 m buffer were higher among children who lived in districts with a low SES (ref. high SES). 

**Table 2:** Distribution of neighbourhood environment variables among children (*n* = 485).

	Predictor variables	Median (IQ) or % (95% CI)
**Built environment**	**House type**	*n* = 481
	Single-unit/semi-detached	66.9 (62.7–71.1)
	Apartment building ≤ four floors	25.8 (21.9–29.7)
	≥five floors	7.3 (5.0–9.6)
	**Garden**	*n* = 469
	Yes/no	91.3 (88.7–93.9)/8.7 (6.1–11.3)
	**Street type**	*n* = 479
	Pedestrian zone or cul-de-sac or play streets	30.1 (26.0–34.2)
	Side street (traffic calmed)	50.1 (45.6–54.6)
	Side street	8.8 (6.3–11.3)
	Main street	11.1 (8.3–13.9)
	**Traffic density ^1^**	
	High/middle/low	23.3 (19.5–27.1)/30.7 (26.6–34.8)/46.0 (41.5–50.5)
	**Perceived frequency of traffic jam**	*n* = 481
	High/middle/low	0.6 (−0.1–1.3)/3.7 (2.0–5.4)/95.7 (93.9–97.5)
	**Perceived frequency of passing trucks and buses**	*n* = 478
	High/middle/low	13.0 (10.0–16.0)/19.0 (15.5–22.5)/68.0 (63.8–72.2)
	**Walkability ^1^**	
	High/middle/low	15.7 (12.4–19.0)/54.6 (50.1–59.1)/29.7 (25.6–33.8)
	**Length of access path ^2^**	20.5 (17.2–23.0)
	**Distance (m) to nearest**	
	Park/green space	495.5 (234.0–912.8)
	Playground	205.8 (128.8–325.1)
	**Restriction by lack of **	
	**Parks/green space**	*n* = 475
	High/middle/low	3.2 (1.6–4.8)/4.2 (2.4–6.0)/92.6 (90.2–95.0)
	**Playgrounds**	*n* = 479
	High/middle/low	4.0 (2.2–5.8)/7.3 (5.0–9.6)/88.7 (85.9–91.5)
	**Cycling paths**	*n* = 475
	High/middle/low	8.2 (5.7–10.7)/9.3 (6.7–11.9)/82.5 (79.1–85.9)
	**Number (n) of ^2^**	
	Supermarkets	2 (0–3)
	Energy-dense food supply	6 (3–11)
**Social environment**	**Neighbourhood SES ^1^**	
	High/middle/low	46.6 (42.1–51.1)/20.4 (16.8–24.0)/33.0 (28.8–37.2)
	**Crime rate ^1^**	
	High/middle/low	8.2 (5.7–10.7)/54.2 (49.7–58.7)/37.5 (33.1–41.9)
	**Neighbourhood safety**	*n* = 471
	Yes/no	93.8 (91.6–96.0)/6.2 (4.0–8.4)
	**Satisfaction with neighbourhood**	*n* = 480
	High/middle/low	81.5 (78.0–85.0)/13.8 (10.7–16.9)/4.8 (2.9–6.7)

SES: socioeconomic status; ^1^ referring to district; ^2^ within an 800 m Euclidian buffer of each participant address point.

As shown in Table 3, socially disadvantaged neighbourhoods were especially inhabited by “non-German” children—independently of their parents’ educational level. Among “German” children, parental education was inversely associated with the neighbourhood SES. Furthermore, families living in neighbourhoods with low SES less often lived in single-unit or semi-detached houses (35.4% *vs*. 82.6%, *p* = 0.000, ref. high SES), more often indicated a high frequency of passing trucks/busses (21.0% *vs*. 9.0%, *p* = 0.000), they more frequently felt unsafe in their district (14.6% *vs*. 0%, *p* = 0.000) and were less often satisfied with their neighbourhood (68.2% *vs*. 89.7%, *p* = 0.000). 

**Table 3:** Estimated probability (%) of living in a neighbourhood (district) with a certain socioeconomic status (SES): interactions between parents’ educational level and nationality.

Nationality	Parents’ educational level	Neighbourhood SES		
		High	Middle	Low
“German”	High	**62.7**	**20.9**	**16.4**
	Middle	**38.4**	**27.0**	**34.7**
	Low	**11.6**	**16.8**	**71.6**
“Non-German”	High	19.6	22.8	57.6
	Middle	12.0	17.3	70.7
	Low	13.7	18.7	67.6

Marked bold: significant differences between groups of district SES (ordinal regression, *p* < 0.001).

### 3.2. Longitudinal Influences of the Neighbourhood Built and Social Environment on Children’s BMI-SDS

Unadjusted and adjusted multivariable associations between children’s BMI-SDS and environmental parameters which were related (*p* < 0.05) with the outcome in the bivariate analysis (Model 1) are shown in Table 4. All four environmental parameters (street type, perceived frequency of passing trucks/buses, walkability and neighbourhood SES) remained significant after entering into the multivariate model (Model 2). However, only neighbourhood SES had an effect on change in BMI-SDS in this study population. Children who lived in socially disadvantaged neighbourhoods had 0.31 units higher BMI-SDS compared to the reference category (high neighbourhood SES). After adjustment for individual and social factors (Model 3), the results were quite similar to Model 2. However, inclusion of interaction terms between time, neighbourhood and parents’ educational level/nationality showed that the association between children’s BMI-SDS over 4 years and neighbourhood SES was partially explained by social characteristics of the family.

## 4. Discussion

The present study demonstrated that aspects of neighbourhood social and built environment were associated with higher BMI-SDS, but only neighbourhood SES had an effect on change of children’s BMI-SDS. However, the full adjusted GEE model revealed that the neighbourhood environment had a limited association with childhood overweight in this study.

### 4.1. Built Environment and Children’s BMI-SDS

In the multivariate analysis, walkability was inversely associated with children’s BMI-SDS, but did not predict change in BMI-SDS over 4 years. This result partially differs from those of Slater *et al*. and Spence *et al*. which showed that living in a high walkable neighbourhood is associated with lower odds of being overweight/obese among children and adolescents [23,24]. 

**Table 4:** Regression coefficients derived from GEE-analysis, investigating the longitudinal relationship between children’s BMI-SDS over 4 years (outcome variable) and neighbourhood built and social environment ^1^. Bivariate and multivariate analysis and multivariate analysis (full model).

				Model 1			Model 2		
				b	95% CI	*p*-value	b	95% CI	*p*-value
**Built environment**	**Time** (ref: baseline)						−0.14	−0.30–0.03	0.101
	**Street type**								
			Pedestrian zone or cul-de-sac or play streets	Ref			Ref		
			Side street (traffic calmed)	**0.27**	0.09–0.44	0.003	**0.23**	0.06–0.40	0.010
			Side street	0.25	−0.07–0.58	0.123	0.12	−0.21–0.44	0.478
			Main street	0.27	0.00–0.54	0.050	0.10	−0.22–0.41	0.539
	**Perceived frequency of passing trucks and buses**								
			Low	Ref			Ref		
			Middle	0.16	−0.03–0.36	0.105	0.11	−0.10–0.32	0.318
			High	**0.40**	0.12–0.69	0.006	**0.34**	0.03–0.65	0.034
	**Walkability ^2^**								
			High	Ref			Ref		
			Middle	**0.53**	0.30–0.75	0.000	**0.49**	0.26–0.73	0.000
			Low	**0.33**	0.10–0.57	0.006	**0.45**	0.19–0.71	0.001
	Time * Walkability								
			Time * Walkability (high)				Ref		
			Time * Walkability (middle)				0.07	−0.11–0.25	0.448
			Time * Walkability (low)				−0.02	−0.21–0.17	0.828
**Social environment**	**Neighbourhood SES ^2^**								
			High	Ref			Ref		
			Middle	**0.44**	0.23–0.65	0.000	**0.23**	0.06–0.46	0.044
			Low	**0.25**	0.07–0.43	0.006	0.06	−0.14–0.26	0.552
	Time * Neighbourhood SES								
			Time * SES (high)				Ref		
			Time * SES (middle)				0.20	0.03–0.36	0.019
			Time * SES (low)				0.31	0.17–0.45	0.000
							**Model 3**		
							**b**	**95% CI**	***p*-value**
**Built environment**	**Time** (ref: baseline)						−0.16	−0.36–0.05	0.135
	**Street type**								
			Pedestrian zone or cul-de-sac or play streets				Ref		
			Side street (traffic calmed)				**0.21**	0.03–0.38	0.020
			Side street				0.03	−0.32–0.37	0.878
			Main street				−0.14	−0.32–0.29	0.927
	**Perceived frequency of passing trucks and buses**								
			Low				Ref		
			Middle				**0.23**	0.02–0.44	0.030
			High				0.17	−0.15–0.49	0.287
	**Walkability ^2^**								
			High				Ref		
			Middle				**0.44**	0.18–0.71	0.001
			Low				**0.45**	0.18–0.72	0.001
	Time * Walkability								
			Time * Walkability (high)				Ref		
			Time * Walkability (middle)				−0.02	−0.22–0.18	0.842
			Time * Walkability (low)				−0.08	−0.29–0.13	0.461
**Social environment**	**Neighbourhood SES ^2^**								
			High				Ref		
			Middle				0.06	−0.22–0.34	0.672
			Low				0.27	−0.03–0.58	0.076
	Time * Neighbourhood SES								
			Time * SES (high)				Ref		
			Time * SES (middle)				**0.28**	0.09–0.47	0.004
			Time * SES (low)				**0.32**	0.11–0.54	0.003
	Time * neighbourhood SES (parents’ educational level (EL))								
			Parents’ EL (high) (Time * SES)				N/A		
			Parents’ EL (middle) (Time * SES)						
			SES (high)				Ref		
			SES (middle)				**0.50**	0.04–0.97	0.033
			SES (low)				−0.26	−0.69–0.16	0.227
			Parents’ EL (low) (Time * SES)						
			SES (high)				Ref		
			SES (middle)				−0.18	−1.36–1.01	0.769
			SES (low)				−0.03	−0.87–0.81	0.941
	Time * neighbourhood SES (parents’ nationality)								
			Nationality (“German”) (Time * SES )				N/A		
			Nationality (“non-German”) (Time * SES)						
			SES (high)				Ref		
			SES (middle)				0.00	−0.69–0.69	0.998
			SES (low)				−0.09	−0.60–0.41	0.719

BMI-SDS: body mass index—standard deviation score; SES: socioeconomic status; ^1^ GEE: Generalized estimating equations; N/A: not applicable; Model 1: bivariate analysis; Model 2: adjusted for all factors significantly (*p* < 0.05) associated with the outcome in Model 1, including interaction terms (*) (*n* = 473); Model 3: adjusted for children’s sex, educational level and nationality of parents, maternal weight status and all factors significantly (*p* < 0.05) associated with the outcome in Model 1, including interaction terms (*) (*n* = 397). ^2^ Referring to district.

High walkability is characterised by high intersection density and a good street network. Therefore, children living in areas with high walkability have access to several different routes to get to school or playgrounds. So, parents are able to choose the safest route which may increase the probability of children’s active commuting to school. In addition, a better access to parks/green spaces (due to short distances) may add to more frequent outdoor playing. Numerous studies have already demonstrated a positive relationship between walkability and/or street network and physical activity among children and adolescents [25,26,27]. On the other hand, districts with high walkability are frequently characterised by a high traffic density. Accordingly, Timperio *et al*. and Jerret *et al*. indicated a high increase in BMI among children and adolescents who lived in neighbourhoods with high traffic density [28,29]. In this study, traffic density had no association with children’s BMI-SDS. However, the perceived frequency of passing trucks or busses was related to BMI-SDS—even though it did not remain significant after including the time interaction term. It can thus be suggested that parents’ perception rather than the objective neighbourhood environment may be decisive. If parents perceive the streets as highly frequented roads, children may have less permission to walk, cycle or to play alone outdoors. 

The number of food outlets was not significantly associated with BMI-SDS over 4 years. These findings are consistent with earlier research [10,30]. However, Oreskovic *et al*. and Jennings *et al*. reported a positive association between availability of unhealthy food outlets and children’s weight status [8,31]. However, future studies on this topic are necessary, because it is unclear whether these food outlets were frequented by children. It is also currently unclear, whether the distance and number of food outlets can affect parents’ purchasing behavior and how this could influence children’s diet and weight status. 

### 4.2. Social Environment and Children’s BMI-SDS

In GEE-analysis, we observed a significant and inverse relationship between neighbourhood SES and change in BMI-SDS. However, taking into account potential interactions between neighbourhood SES and parental educational level, neighbourhood SES had only an effect on change in BMI-SDS among children whose parents had a middle educational level. This finding does not support previous data from Germany, Spain or Canada: Igel *et al*., Navalpotro *et al*. and Oliver and Hayes indicated that neighbourhood SES is independently related to the weight status of children [16,32,33,34]. However, these studies had a cross-sectional design or used different characteristics of the neighbourhood SES. Furthermore, social neighbourhood does not primarily affect children’s BMI-SDS directly. This relationship is mediated by children’s physical activity and diet. Thus, future research has to use path analysis/structural equation models to analyse these relationships and to avoid problems of multicollinearity.

Powell *et al*., Moore *et al*. and Schneider and Gruber demonstrated that neighbourhoods with low SES provided fewer opportunities for physical activity (e.g., low availability of parks/green space), but more food outlets with “unhealthy” food [35,36,37]. By contrast, in this study, distances to the nearest park and playground or number of outlets selling energy-dense food were not associated with district SES, whereas parents’ perceived neighbourhood safety and frequency of passing trucks/busses had a significant association with district SES. These data suggest that the health-related effects of the neighbourhood built and social environment are complex and multidimensional; in addition, these effects may differ among countries or cities. For this reason, neighbourhood disadavantages should be characterised individually for each city. Regarding preventive activities, this idea suggests that specific local policy initiatives have to be developed to reduce neighbourhood disadvantage. 

### 4.3. Limitations

In this paper, it was not possible to additionally adjust for clustering within neighbourhoods, because we have used two different ways to define neighbourhoods. Most parameters were based on district data, but we have measured the number of food outlets within an 800 m buffer of participant’s home, because the use of administrative units to define neighbourhoods may not reflect children’s true neighbourhood exposure level. Furthermore, the food environment and parents’ perception of their neighbourhood environment were assessed at follow-up only. While the neighbourhood environment is likely to be relatively static, parents’ perception may have changed over time. 

## 5. Conclusion

Compared to the neighbourhood built and social environment, familial/social factors have a greater impact on children’s BMI-SDS over 4 years. Thus, social inequalities in children’s health (regarding to overweight) are partially explained by social neighbourhood characteristics only. Nevertheless, the present data provide some evidence for future strategies of health promotion and prevention taking into account individual and environmental determinants of childhood health.
